# Supplementary material for: The interactome of intact mitochondria by cross-linking mass spectrometry provides evidence for coexisting respiratory supercomplexes
Source: Mol Cell Proteomics. 2017 Dec 8;17(2):216–32. doi: 10.1074/mcp.RA117.000470 (PMC5795388; doi:10.1074/mcp.RA117.000470)
Supplement: Supplemental Data [file supp_RA117.000470_133922_0_supp_23978_fzffwf.zip › spectra_annotation/mito_spectra_annotation/165-1-5-1-21-1.pdf]

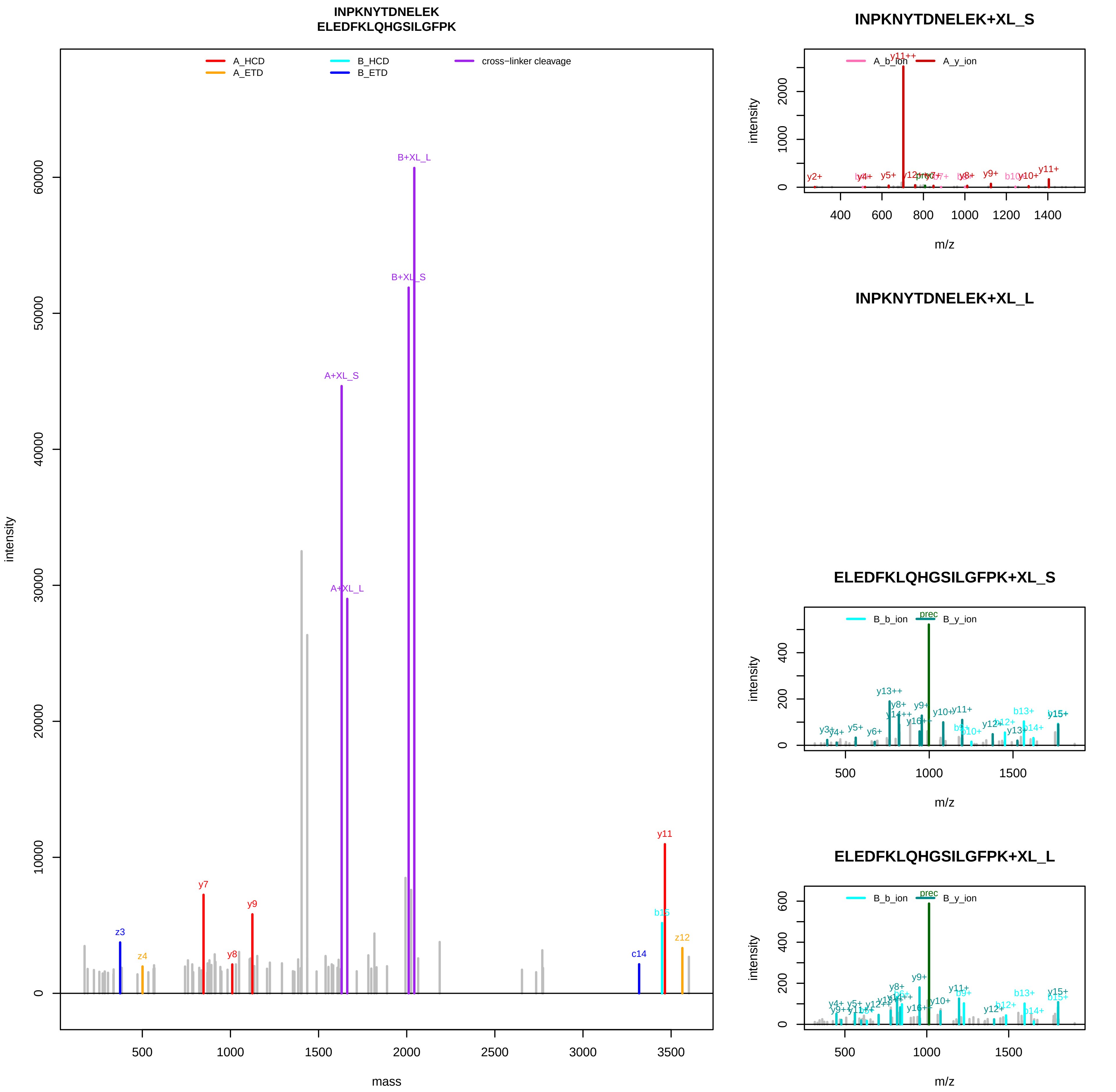
INPKNYTDNELEK
ELEDFKLQHGSILGFPK
A_HCD
A_ETD
B_HCD
B_ETD
cross−linker cleavage
B+XL_L
B+XL_S
A+XL_S
A+XL_L
z3
z4
y7
y8
y9
c14
b15
y11
z12
intensity
mass
INPKNYTDNELEK+XL_S
A_b_ion
A_y_ion
y11++
y2+
y4+
y5+
y9+
y10+
y11+
m/z
INPKNYTDNELEK+XL_L
ELEDFKLQHGSILGFPK+XL_S
B_b_ion
B_y_ion
prec
y13++
y15+
b13+
ELEDFKLQHGSILGFPK+XL_L
